# Identifying and Characterizing Regulatory Sequences in the Human Genome with Chromatin Accessibility Assays

**DOI:** 10.3390/genes3040651

**Published:** 2012-10-15

**Authors:** Nathan C. Sheffield, Terrence S. Furey

**Affiliations:** 1 Program in Computational Biology and Bioinformatics, Institute for Genome Sciences & Policy, Duke University, Durham, NC 27708, USA; E-Mail: nathan.sheffield@duke.edu; 2 Depts of Genetics and Biology, Carolina Center for Genome Sciences, Lineberger Comprehensive Cancer Center, University of North Carolina, Chapel Hill, NC 27599, USA

**Keywords:** open chromatin, DNaseI, transcriptional regulation, ENCODE

## Abstract

After finishing a human genome reference sequence in 2002, the genomics community has turned to the task of interpreting it. A primary focus is to identify and characterize not only protein-coding genes, but all functional elements in the genome. The effort includes both individual investigators and large-scale projects like the Encyclopedia of DNA Elements (ENCODE) project. As part of the ENCODE project, several groups have identified millions of regulatory elements in hundreds of human cell-types using DNase-seq and FAIRE-seq experiments that detect regions of nucleosome-free open chromatin. ChIP-seq experiments have also been used to discover transcription factor binding sites and map histone modifications. Nearly all identified elements are found in non-coding DNA, hypothesizing a function for previously unannotated sequence. In this review, we provide an overview of the ENCODE effort to define regulatory elements, summarize the main results, and discuss implications of the millions of regulatory elements distributed throughout the genome.

## 1. Introduction

The sequencing of the human genome has both facilitated progress and uncovered new challenges. The sequence itself is a trove of data benefiting diverse biological disciplines. Insights enabled by the human genome project are fueling the drive toward personalized medicine and impacting the diagnosis and treatment of human disease [1]. Though our knowledge has increased considerably, there are many unanswered questions. Foremost is the task of converting so much data into meaningful information. With the genome sequence in hand, how do we make sense of what it says? Assigning even a basic function for the majority of the genome has still not been completed. Despite monumental progress, a primary goal remains to determine *what the genome actually encodes*.

This is not a new goal. Attempts to discover the functional “meaning” of the whole human genome sequence dates to before the sequencing project began. About 40 years ago, with preliminary evidence, Ohno suggested that only 6% of the human genome consists of genes and promoters [2]. He was not far off—more recent estimates put that number near 2–3% [3,4]. Since Ohno’s proposal, the community has debated the function of the remaining 94%+ of the genome: is it “junk DNA” [3]? Simply decoding the complete sequence has not answered the question, partly because non-coding DNA lacks the information-rich genetic code that has made identifying protein-coding sequences possible. However, the community has now amassed experimental evidence that much noncoding, intergenic DNA is important for transcriptional regulation. These sequences regulate transcription by controlling when a gene is expressed with respect to, for example, cell-type, developmental stage, or environment. It is still possible that some of the genome does not have a direct cellular function, such as particular repetitive sequences that have not yet been thoroughly explored [4], but it is clear that much of the genome is regulatory. This has led to a multifaceted effort to identify and characterize the regulatory sequences in the human genome.

**Figure 1:**
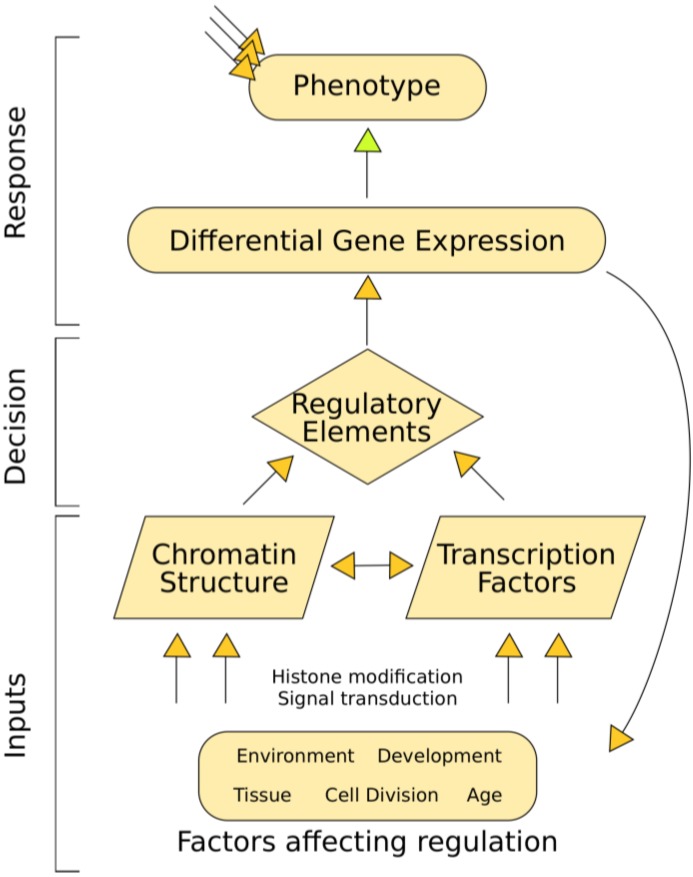
Transcriptional regulation flowchart showing how regulatory elements convert input information into phenotype. A variety of internal and external factors alter chromatin structure and transcription factor binding. These regulatory inputs control the availability of regulatory elements like enhancers, silencers, or promoters. Regulatory elements drive gene expression, which ultimately control phenotype (along with other regulatory influences like RNA decay).

Cataloging regulatory elements is vital for a complete understanding of human biology. Like all multicellular organisms, humans are composed of a diverse set of cell-types with widely divergent phenotypes that interact in complex ways. The diversity of cellular phenotype is possible because of differential gene regulation, despite cells having identical genomes. Each cell-type activates different genes via appropriate regulatory elements that encode instructions dictating a cell's response to both external and internal stimuli. In an information flowchart, regulatory elements could be considered decision-making entities that transfer input information into the organism's response (Figure 1). A difference in phenotype or response to stimulus is often driven by differences in gene expression, which are in turn governed by regulatory elements. Thus, regulatory element activity can be viewed as a genome-based signal that drives differences in phenotype. In order to piece together how humans work, we must understand the differences among cell-types, including decoding their specific regulatory elements.

Thanks to recent biotechnology advances, we can now examine regulatory sequences in depth genome-wide. Several methods have recently been applied to genome-wide regulatory element study [5], including the DNaseI hypersensitivity assay (DHS) [6,7], formaldehyde-assisted isolation of regulatory elements (FAIRE) [8], and chromatin immunoprecipitation (ChIP) [9]. Each of these experiments has advantages and disadvantages. The ENCODE consortium [10] has recently generated massive amounts of data from each of these experiments. To complement this data, it has been and will continue to be necessary to develop computational algorithms and tools to aid in its interpretation. Such data and analysis have the potential to help us understand how chromatin structure contributes to transcription factor binding, gene expression, and ultimately phenotypic differences. In this review, we provide an overview of the ENCODE effort to define regulatory elements based on these experiments, summarize the general results, and discuss implications of the millions of regulatory elements distributed throughout the genome.

## 2. Background

### 2.1. Chromatin Structure

**Figure 2:**
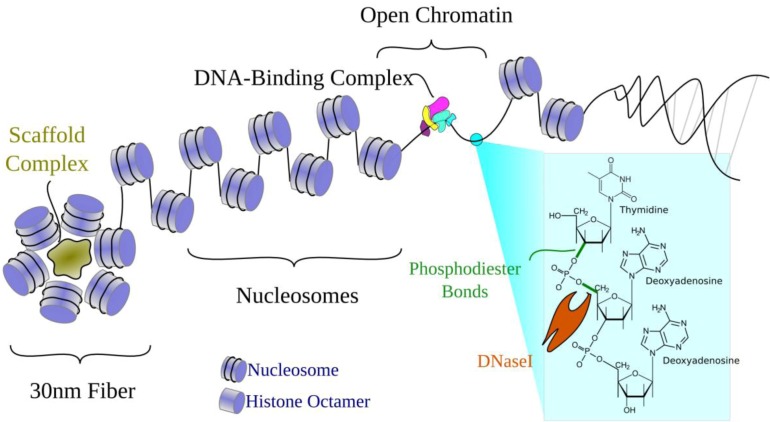
Open chromatin and the organization of DNA in a cell. DNA is tightly wound around histone proteins to form nucleosomes. Nucleosomes are further wrapped around scaffold proteins to form the 30 nm fiber. In unwrapped regions where histones are displaced, the DNA is open for recognition by transcription factors. Open chromatin regions are also susceptible to DNaseI, which cleaves phosphodiester bonds in the DNA backbone to sever DNA molecules.

Chromatin structure has long been known to affect tissue-specific transcriptional regulation [11,12]. Figure 2 illustrates the familiar organization of DNA in the genome. Briefly, the double helix wraps around histone protein octamers to form nucleosomes that then, with additional scaffold proteins, form higher-order 30 nm fibers. Transcriptionally silenced regions are generally packaged into tightly-packed heterochromatin. Actively transcribed genes typically remain in the more loosely-packed euchromatin, where DNA is more accessible to the transcriptional machinery. Within euchromatin, some stretches of DNA are less associated with histones. These "unwrapped" regions are referred to as open, accessible, or nucleosome-depleted regions. Nucleosome-depleted regions can interact with DNA-binding proteins, which can then regulate nearby chromatin structure and gene expression.

Thus, one avenue for chromatin structure to affect transcriptional regulation is through open chromatin being bound by sequence-specific transcription factors. Scientists have been studying regulatory elements using experiments that locate open chromatin since the 1970s [13]. However, despite the realized importance and interest in studying regulatory DNA, initial studies of the human genome sequence instead focused on protein-coding genes. This is partly due to difficulties with noncoding DNA: Identifying and assigning functions to regulatory elements is complicated by several issues.

### 2.2. Challenges to Studying Regulatory Elements

What makes studying regulatory elements so difficult? First is the sheer number of elements; there are far more than genes. Our initial estimate of 30,000 to 40,000 genes in the human genome [4] has more recently been reduced to 20,000–25,000 [14]. In contrast, the number of proposed regulatory elements currently stands in the millions and continues to rise [15,16]. This complexity has given rise to the term “regulome,” symbolizing the growing set of regulatory components. The fragmentation and volume of regulatory sequence (perhaps one-third of the genome *vs*. 2% being coding regions) makes it difficult to study [16]. 

Second, they are difficult to find. Unlike protein-coding genes, which have a rigid genetic code, regulatory elements seem to lack such a rigid code and are thus more difficult to identify computationally. Where regulatory elements do follow patterns, such as conforming to transcription factor binding motifs, they are more elastic, which has led to more sequence variation in regulatory elements than in protein-coding genes. Sequence conservation yields some clues (e.g., [17]), but active functional elements may not be under detectable evolutionary constraint [18,19]. For this reason, we must currently rely mainly on experimental methods to identify regulatory elements.

Third, after identifying regulatory elements, further difficulties arise in determining their targets [20]. Regulatory elements can act both directly and indirectly to modulate transcriptional levels. For direct (or *cis-*) regulation, target genes are not obvious because an element does not necessarily regulate the single nearest gene; instead, they can act at large distances [21], skip genes [22], or affect multiple genes [23]. Elements can also act indirectly (in *trans-*) to affect genes *en masse* by regulating a transcription factor with many targets. These layers form the robust system required to thrive in a changing environment, but they are not easy to dissect and explain.

Fourth, regulatory elements have a variety of functions [24]. For example, there are distinctions between promoters, enhancers [25], enhancer blockers [26], insulators [27], LCRs [28], and Polycomb-bound silencers [29]. One step further, within each category there may be additional subdivisions, such as the distinction between TATA-box *vs*. CpG-rich promoters [30] or enhancer sub-classes [25]. Each performs a different but necessary purpose, but the sequence alone is currently unable to classify the elements by type. Computational research continues to identify subtle sequence patterns [31], but our understanding of sequence remains a key limitation. 

Finally, perhaps the most important challenge to studying transcriptional regulation is that the regulome is dynamic. The human regulome varies in many dimensions, such as age, environment, developmental time, cell-cycle stage, and tissue type. In contrast, the genome is essentially constant and identical in each cell and tissue type. To identify every regulatory element in the human genome is a currently infeasible task; it would require interrogating every cell-type under every possible developmental stage in any environment and against all genetic backgrounds.

Despite these challenges, considerable recent progress has been made toward identifying and characterizing regulatory elements. Several of these difficulties have only started to become tractable in the past few years due in large part to technological improvements in sequencing and computation, which have driven new discovery in almost every biological field. In the study of transcriptional regulation, the major genome-wide findings have been primarily driven by results from chromatin accessibility and transcription factor experiments that assay regulatory elements.

### 2.3. Open Chromatin and Regulatory Element Assays

Three common experimental techniques used to assay chromatin structure and identify regulatory elements are DNaseI hypersensitivity (DHS), formaldehyde assisted identification of regulatory elements (FAIRE), and chromatin immunoprecipitation (ChIP). These assays each have strengths and weaknesses.

#### 2.3.1. DNaseI Hypersensitivity (DHS)

Scientists have long used DNaseI hypersensitivity (DHS) assays to distinguish between open regions of DNA and those protected from digestion by nucleases [32]. Deoxyribonucleases (DNases) are enzymes that cleave phosphodiester bonds in DNA (Figure 2). There are several types of DNases, including restriction enzymes, which are sequence-specific DNases. In contrast, DNaseI cleaves non-specifically (without sequence preference). DNaseI is a fairly large enzyme, which limits its ability to penetrate tight spaces and restricts it to cleaving DNA that is easily accessible. In a DNaseI hypersensitivity experiment, DNA is treated with a small concentration of DNaseI. The cuts can then be located within the genome and quantified to annotate open chromatin. In the original experiments, this was done on individual loci and cuts were mapped using the electrophoretic separation of radiolabelled digested fragments on polyacrylamide gels. Now, we use high-throughput sequencing to interrogate digestion genome-wide with greater resolution [6]. As a result, after sequencing short DNA molecules corresponding to DNaseI cuts and aligning these to a reference sequence, we get a profile of “open” regions in the genome. Because transcription factors also tend to bind in such open areas, DNA that is accessible to DNaseI primarily corresponds to regulatory elements. Since many (probably most) changes in the accessibility of DNA are associated with regulatory processes [33], DNaseI assays have been the gold standard in locating regulatory elements. One particular advantage of DNaseI experiments is that they can detect all types of active elements, even without prior knowledge of function [34]; however, they do not identify what specific factors bind there. They simply distinguish between open and closed DNA.

#### 2.3.2. Formaldehyde-assisted Isolation of Regulatory Elements (FAIRE)

FAIRE is a more recent technique that highlights similar open chromatin regions [8]. It is a relatively simple experiment that involves only a few steps: first, a formaldehyde step to fix protein-DNA interactions, followed by sonication to fragment the genome, and finally a phenol-chloroform extraction to separate bound from unbound DNA. Unbound DNA is then sequenced and aligned to identify nucleosome-depleted regions. The results overlap considerably, but not completely, with DNaseI regions [35]. The advantages of FAIRE are that it is highly reproducible and that the samples require relatively minimal preprocessing, reducing potential artifacts and enabling the experiment to be done on a variety of sample types [8]. However, the final signal is more diffuse and lacks additional information used for fine-resolution DNaseI footprint mapping [36,37].

#### 2.3.3. Chromatin Immunoprecipitation (ChIP)

Like DHS assays, ChIP dates from several decades ago [38]. In ChIP, like FAIRE, a lysate is cross-linked to fix protein-DNA reactions, and sonicated or digested to shear the genome. Then, antibodies are used to “pull down” a particular protein of interest, cross-links are reversed, and DNA is sequenced. Two common uses of ChIP are 1) to find where *specific factors* bind, and 2) to identify histone tail modifications. In the first case, ChIP differs from DHS and FAIRE assays in that it targets a specific factor. This is both an advantage and a disadvantage; the factor bound is revealed, but it requires specific antibodies and individual experiments for each factor of interest. Thus, we can only use ChIP on factors that are known *a priori*. In the second case, ChIP uses antibodies against histone proteins (rather than transcription factors), with different antibodies targeting histones with different chain modifications. This type of ChIP experiment does not identify individual transcription factors, but nucleosomes with particular modifications. This usually results in a much more diffuse signal covering multiple nucleosomes; it does not identify specific TFs but it does give a clue as to the function of the region because some modifications have been associated with certain types of regulatory functions [39].

There have been several major advances in ChIP technology recently; most notable is ChIP-seq, the combination with sequencing to look at genome-wide TF binding [9]. ChIP has also been recently modified to require fewer cells [40], include methylation status [41], and provide better resolution with exonucleases [42]. One of the key remaining limitations with ChIP is the availability of high quality antibodies [43].

#### 2.3.4. Other Similar Assays

MNase-seq is similar to DNase-seq, but replaces DNaseI with MNase (micrococcal nuclease) [44]. The principles of the experiment are the same, but MNase is a smaller molecule than DNaseI, enabling it to digest smaller, less accessible areas. It is able to cleave the linker region between nucleosomes. It also has exonuclease activity, so it digests back from the initial cuts until it reaches a bound protein protecting the DNA. Rather than sequencing where the cuts are, this method sequences the DNA left intact, indicating the location where something (a nucleosome or transcription factor) is bound. This can highlight exact nucleosome positioning, but it requires deeper sequencing because it sequences the inverse regions: locations of nucleosomes, rather than open regions (less than 5% of a genome is open in any given DHS experiment [35]). For this reason, it has primarily been used in smaller genomes, like yeast, but a limited amount of data from human is also available [44]. Other similar techniques include Sono-seq [45] and Nome-seq [46] which also identify open chromatin; Sono-seq uses sonication instead of DNaseI to fragment DNA, while Nome-seq uses a methyltransferase to mark regions of open chromatin with GC methylation.

One key limitation of all of the above methods is that the regions they identify are only *potentially* functional. Open chromatin, and even evidence for a bound transcription factor, implies but does not demonstrate regulatory potential [24]. It is possible for regions to be open or even bound but still lack regulatory effect. Reporter assays are commonly used to validate function, but currently these are done on an individual-site basis, limiting the number of sites that can be reasonably tested.

## 3. Identifying and Characterizing Regulatory Elements

The most common experiment among those mentioned above has been ChIP. Several groups in the ENCODE Consortium have published the results of hundreds of ChIP experiments for different cell-types and transcription factors [10,47]. Recent reviews have summarized these results [48,49]. In this review we focus on recent work identifying regulatory elements using DNaseI hypersensitivity and FAIRE in the ENCODE project. This work has both confirmed hypotheses formed on smaller samples and has revealed new findings regarding how transcriptional regulation works at the genomic level [16,35].

### 3.1. Open Chromatin Defines Regulatory Elements

The working hypothesis of the past several decades has been that open chromatin identifies regulatory regions. Genome-wide results now confirm this finding: Thurman *et al*. [16] showed that the DNaseI-seq profile recapitulates the sum of TF ChIP-seq signals. In K562 cells, with ChIP-seq results available for more than 42 factors, the correlation between the cumulative ChIP results and DNaseI-seq is very high (~0.8). Almost 95% of known ChIP-seq peaks are in regions identified as open by DNaseI assays. This result shows that open chromatin is a reasonable proxy for generic TF binding and highlights the utility of open chromatin assays.

### 3.2. Regulatory Elements are Located in Promoter, Intergenic, and Intronic Regions

**Figure 3:**
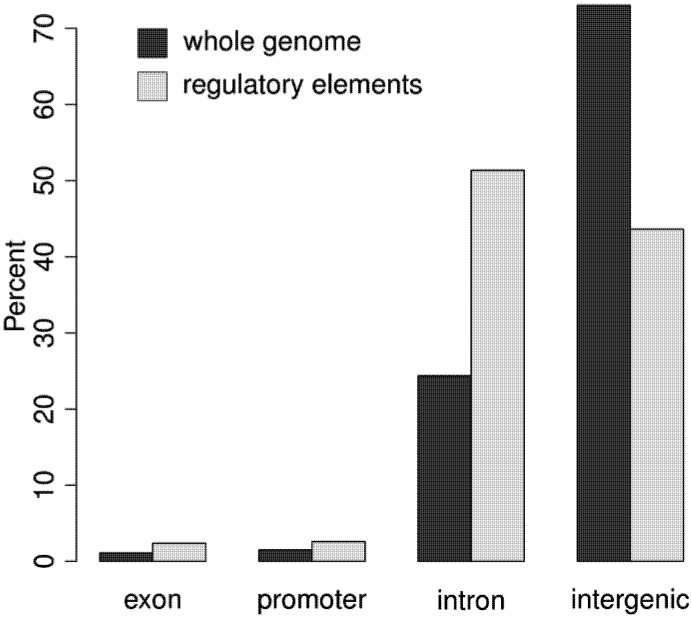
The distribution of the genome as a whole compared to the distribution of regulatory elements with respect to known transcribed regions. Dark bars reflect the genomic distribution while the light bars reflect the distribution of regulatory elements in four categories: exon, promoter, intron, and intergenic regions. Regulatory elements are located near (promoters) or within (introns, exons) known genes at greater frequency than would be expected given the genomic distribution.

The distribution of DNA in the genome relative to known transcribed regions is shown in Figure 3, and compared to the distribution of regulatory elements. The vast majority of regulatory elements are noncoding, with about 5% identifying known promoter elements, and the other noncoding elements almost evenly split between intergenic and intronic sequences [16]. There are some rare exonic open chromatin regions, which could regulate splicing [50], or they may overlap intronic elements. The clear emphasis on distal regulation (most elements are intergenic or intronic) supports the growing realization that distal elements are a key source of phenotypic complexity [25].

### 3.3. More than 30% of the Genome May be Regulatory

**Figure 4:**
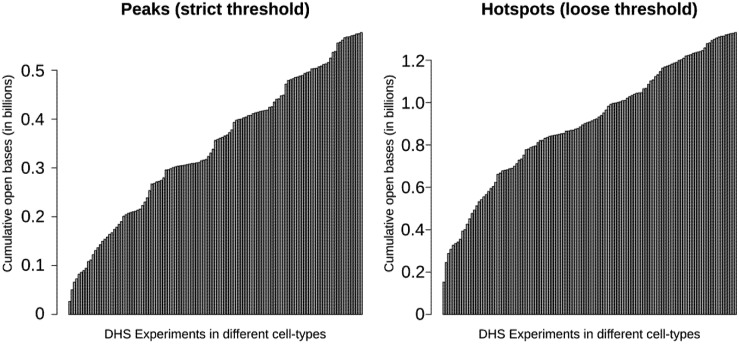
The cumulative number of bases contained in DNaseI-hypersensitive sites according to annotations by Thurman *et al*. (2012). This plot shows how the total number of accessible bases in the genome (y-axis) grows as additional samples (x-axis) are considered. Starting with a single sample on the left, the cumulative number of accessible bases is calculated by taking the union of the DHS sites from each successive sample. Two different threshold levels are shown, “peaks” (hypersensitive) and “hotspots” (more generally sensitive). Ultimately, greater than 1.2 billion nucleotides are annotated as within a DHS hotspot, with over 500 million nucleotides locating within a DHS peak.

Using a sample of 126 cell types, Thurman *et al*. [16] reported that nearly one-third of the genome shows sensitivity to DNaseI digestion with ~15% of the genome being DNaseI hypersensitive. These percentages differ depending on the threshold used to define hypersensitivity, but they provide an idea about the overall proportion of the genome that is sensitive to DNaseI. These numbers should be considered minimums, as they are restricted to the cell-types, environments, and developmental stages assayed. Figure 4 shows how the number and percentage approaches saturation with increasing cell-types. This trend is not simply a result of false-positive DNase-seq signal, which is estimated to be less than 0.5% [16]. The numbers will grow as new cell-types and contexts are assayed. Without testing all cell-types and contexts, the exact percentage of regulatory DNA in the human genome cannot be determined, but these results demonstrate that it may be quite high. Ultimately, these regions will have to be tested to establish function.

### 3.4. Most Regulatory Elements are Cell-Type-Specific

With open chromatin data from a variety of cell-types, it is possible to classify regulatory elements as cell-type-specific, shared among multiple cell-types, or ubiquitous. This cell-type-specificity classification necessarily depends on the cell-types used. Many elements currently considered cell-type-specific may turn out to be present in several related but not yet assayed cell-types. Nevertheless, highly cell-type-specific sites are likely to retain that feature, though it may be adjusted from “cell-type-specific” to “narrow-cell-lineage-specific.” 

Gaulton *et al*. [51] identified thousands of pancreatic-islet-specific FAIRE sites. Song *et al*. [35] used both FAIRE and DNaseI to show similar results in seven other cell-types. These results have illustrated that each cell-type is likely to have a unique set of specific open chromatin regions that guide specific cell fates and functions, in addition to those shared by other cell-types. Both of these studies made two additional observations in relation to cell-type-specificity: First, the cell-type-specific DHS sites were associated with cell-type-specific gene expression; and second, cell-type-specific regulatory elements tended to cluster with respect to genomic location.

Most recently, Thurman *et al*. [16] showed that the majority of DHS sites are found in relatively few cell-types, with the distribution depending on the genomic context of the DHS site. For example, promoter DHS sites were more likely to be ubiquitous rather than cell-type-specific.

### 3.5. Transcription Factor Binding Affects Chromatin Structure

One way a cell can affect cell-type-specific expression is by creating a cell-type-specific chromatin conformation. It is now becoming clear that a large class of regulatory elements is involved in establishing cell-type-specific chromatin structure. For example, CTCF sites have many roles affecting chromatin accessibility [52]; CTCF is the canonical insulator, but it also can create both active and repressive loops [53]. Recent studies have shown that other factors also work by altering chromatin structure. Biddie *et al*. [54] showed that DNaseI-hypersensitive sites, specifically with AP1 binding sites, predefine binding sites for GR receptor binding in glucocorticoid cells. Along the same lines, Shibata *et al*. [55] associated AP1 motifs with DNaseI signal differences across primates. Chromatin looping is also likely to be regulated by other factors, such as mediator, p300, and cohesin, which work together to establish cell-type-specific chromatin structure [56]. These results collectively illustrate the important interaction between transcription factors and chromatin structure.

### 3.6. Chromatin Structure Affects Gene Expression

A connection between transcription factor binding and chromatin structure represents one way a cell could regulate gene expression. Recently, Degner *et al*. [57] drew the connection between chromatin and expression in a study that used matched DNaseI-seq, genotype, and expression data from a common cell-type in 70 individuals. They identified about 9,000 sites where DNaseI signal correlated with genotype, and further showed that, in many cases, the DNaseI difference also correlates with gene expression. In a cross-species comparison, Shibata *et al*. [55] showed that DNaseI differences among primates are closely tied to expression differences. These results illustrate how this data may be able to inform computational models to predict gene expression, which has been an area of interest in other organisms [58,59,60]. Along these lines, Natajaran *et al*. [61] built a model to predict gene expression from motif analysis and DNaseI signal. They showed that performance improves when distal elements are included in the model, highlighting the relevance of distal regulatory sites. These results are beginning to unravel the complex interactions between chromatin structure and gene expression.

### 3.7. Regulatory Elements can be Classified by Factor, Function, or Cell-Type-Specificity

One of the most important ways to annotate a regulatory element after discovery is to determine what factors bind there. The primary experimental tool for such an annotation is ChIP. In the ENCODE project, data for hundreds of ChIP experiments have already been made available on the UCSC genome browser [47]. These data enable us to identify the frequency and importance of individual factors [62,63,64,65], as well as explore cooperativity among factors [66]. DNaseI footprinting has also been used to propose what factors bind an element [36,37]. These analyses require not only experimental data, but appropriate computational algorithms to extract meaningful signal.

Regulatory elements can also be divided into functional classes (*i.e.*, silencers, promoters, enhancers). Some classes, like promoters, are relatively straightforward to positionally and functionally define: they lie just upstream of genes and operate on the adjacent gene. Others, like distal enhancers, seem to be anywhere and act on anything. In general, all functional classes share the one major property that they tend to be found in regions of open chromatin. However, they vary in other properties, such as TF binding and histone marks [67]. For example, insulators are often bound by CTCF, whereas enhancers are commonly characterized by certain histone marks (H3K4me1 and H3K27ac) or cofactors (p300) [39]. Quantifying histone marks therefore gives us a relatively straightforward way to provide an initial annotation of regulatory elements genome-wide.

The ENCODE Consortium and others have been performing ChIP-seq to identify multiple histone modifications in several human cell-types. Heintzman *et al*. [39] showed that enhancer histone marks are more often cell-type-specific, in contrast to promoter and insulator marks that are fairly consistent across cell-types. As histone mark data have become available from more cell-types, it has become possible to extrapolate to elements of unknown class and improve current genome annotation. On the basis of this type of data, computational researchers are designing algorithms to characterize regulatory elements. For example, Ernst and Kellis [68] designed a Hidden Markov Model to assign categories to genomic elements on the basis of their histone modification signatures. Lee *et al*. [31] trained a Support Vector Machine to predict mammalian enhancers from genomic sequence and validated the predictions with ChIP and DNaseI data. Classifying regulatory elements by function will be a vital step in understanding transcriptional regulation.

A complementary way to characterize regulatory elements is by cell-type specificity, rather than by experimental signature. Thurman *et al*. [16] used a self-organizing map (a machine learning method) to classify regulatory elements based on their open chromatin signal pattern across all cell-types. They showed that regulatory elements cluster into a limited number of similar patterns. These kinds of classifications will help us derive more meaningful annotations of regulatory elements. By grouping elements by patterns, we may be able to leverage information across open chromatin sites to better annotate how they act. For instance, we have discovered that many groups of DHS sites with similar patterns of accessibility across cell types share common transcription factor motifs suggesting similarly bound factors.

### 3.8. Perspectives

Open chromatin experiments have clearly made progress toward their primary goal to identify regulatory elements, but identifying them is only the beginning. Regulatory elements are dynamic; they drive expression specific to tissue, developmental stage, genetic background, and environment. Distal elements in particular often act in very specific contexts. To decode the human genome, regulatory elements must not only be identified, but also characterized by context specificity. We have described some initial efforts and results in these endeavors; for example, the open chromatin experiments described in this review help us characterize regulatory elements by cell-type. By combining this with ChIP for histone modifications and transcription factors, we can hypothesize the general function of each element; however, we have only begun to assign precise functions. To reveal developmental specificity of an enhancer requires a developmental experiment, such as an embryonic assay in non-human model organisms, accomplished by tethering enhancer sequences to basal promoters [69]. These assays are currently restricted to only certain classes of regulatory elements, and this is an area of active research. In addition, we have little data for environmental context and allelic background [70]. Collecting data from all these sources of variability is a challenge, but to completely understand even a single element, we must characterize it in each context. 

Another key is the question of what genes a particular regulatory element affects. Neither open chromatin, nor ChIP, nor even embryonic assays directly answer this question. The current best methods to reveal gene targets are chromatin conformation capture [71,72] or ChIA-PET [73] experiments. These experiments examine the physical proximity of promoters to regulatory elements. This idea is based on the mounting evidence that physical proximity is associated with regulation [74]; however, its precise importance remains unclear [75]. In addition, experiments that assay the nuclear organization of the genome remain expensive, time-consuming, and difficult to interpret [76]. A potential alternative is to leveraging information across cell-types to correlate cell-type- and developmental-specificity of distal elements with promoter elements [16] or gene expression [77]. However, this method provides only indirect evidence for regulation. Additional research and new methods will be necessary before we will be able to accurately identify the targets of regulatory elements.

Although we still have much to learn, the past 40 years have seen phenomenal advances in our understanding of how the human genome works. These advancements have been driven by the combined effort of both experimental and computational research. Already, there are clear benefits and insights derived from our study of transcriptional regulation, as well as other levels of gene regulation, including mRNA splicing, dispersion, and decay. As we improve our annotations of regulatory elements, our goal to convert this data into information will be realized, ultimately to find the function (if present) of what we once called junk DNA.
